# Exploring an Audio‐based Approach for Early Detection of Alzheimer’s Disease using Chinese Speech Data

**DOI:** 10.1002/alz.088814

**Published:** 2025-01-03

**Authors:** Hung‐Wei Lee, Chia‐ Fang Cheng, Yi‐Hsuan Wang, Ya‐Ning Chang

**Affiliations:** ^1^ Miin Wu School of Computing, National Cheng Kung University, Tainan Taiwan; ^2^ College of Medicine, National Cheng Kung University, Tainan Taiwan

## Abstract

**Background:**

Alzheimer’s disease (AD) has been associated with speech and language impairment. Recent progress in the field has led to the development of automated AD detection using audio‐based methods, because it has a great potential for cross‐linguistic detection. In this investigation, we utilised a pretrained deep learning model to automatically detect AD, leveraging acoustic data derived from Chinese speech.

**Method:**

Speech samples from a picture description task were obtained from 81 native Chinese speakers in Taiwan. This group included 34 normal controls (NC) (14 females; age range: 61‐89 years; average age: 73.9 years; SD: 6.7) and 47 patients diagnosed with early AD (24 females; age range: 59‐89 years; average age: 76.8 years; SD: 7.6). The audio data were first segmented into 6‐second clips, resulting in a total of 1117 clips for the NC group and 1770 for the AD group. Due to the data imbalance, we equalized the groups by randomly selecting 1117 clips from the AD group. The dataset was then divided into a training‐to‐testing ratio of 8 to 2. The training clips were initially processed using a pre‐trained Wav2vec2 model to generate internal acoustic representations. Subsequently, these acoustic representations, now serving as feature data, were input into a two‐layer fully connected neural network for additional training and classification.

**Result:**

The model achieved a training accuracy of 83%. Notable metrics for test performance were observed as follows: Accuracy ‐ 81.25%, Precision ‐ 80.92%, Recall ‐ 81.79%, and F1 Score ‐ 81.35%. These findings suggest a promising capability of wav2vec2 in Alzheimer’s Disease detection, demonstrating a commendable balance between precision and recall.

**Conclusion:**

The pre‐trained Wav2vec2 model emerges as a promising tool for AD detection through Chinese speech data. This research sets the foundation for additional investigation into the model’s potential for cross‐linguistic detection using speech data. Additionally, future work can also be conducted to investigate what acoustic features are used and their significances in classification process.

